# Molecular Epidemiology of SARS-CoV-2 Detected from Different Areas of the Kandy District of Sri Lanka from November 2020–March 2022

**DOI:** 10.3390/v17091189

**Published:** 2025-08-29

**Authors:** Bushran N. Iqbal, Sibra R. M. Shihab, Tao Zhang, Aadhil Ahamed, Shiyamalee Arunasalam, Samanthika Jagoda, Leo L. M. Poon, Malik Peiris, Faseeha Noordeen

**Affiliations:** 1 Diagnostic and Research Virology Laboratory, Department of Microbiology, Faculty of Medicine, University of Peradeniya, Peradeniya 20400, Sri Lanka; iqbalbushran@gmail.com (B.N.I.); shibrashihaab@gmail.com (S.R.M.S.); aadhilahamed40@gmail.com (A.A.); shiyamaleearunasalam@gmail.com (S.A.); 2 School of Public Health, Li KaShing, Faculty of Medicine, The University of Hong Kong, Hong Kong SAR, China; zhangtao@hku.hk (T.Z.); llmpoon@hku.hk (L.L.M.P.); malik@hku.hk (M.P.); 3 Department of Veterinary Pathobiology, Faculty of Veterinary Medicine and Animal Science, University of Peradeniya, Peradeniya 20400, Sri Lanka; samanthika@vet.pdn.ac.lk

**Keywords:** Sri Lanka, Kandy district, SARS-CoV-2 infection, mutation profile, phylogenetic analysis

## Abstract

A comprehensive analysis of the molecular epidemiology of SARS-CoV-2 in the Kandy District of Sri Lanka from November 2020 to March 2022 was conducted to address the limited genomic surveillance data available across the country. The study investigated the circulating SARS-CoV-2 lineages, their temporal dynamics, and the associated mutational profiles in the study area. A total of 280 SARS-CoV-2-positive samples were selected, and 252 complete genomes were successfully sequenced using Oxford Nanopore Technology. Lineage classification was performed using the EPI2ME tool, while phylogenetic relationships were inferred through maximum likelihood and time-scaled phylogenetic trees using IQ-TREE2 and BEAST, respectively. Amino acid substitutions were analyzed to understand lineage-specific mutation patterns. Fifteen SARS-CoV-2 lineages were identified, and of those B.1.411 (36%) was the most prevalent, followed by Q.8 (21%), AY.28 (9.5%), and the Delta and Omicron variants. The lineage distribution showed a temporal shift from B.1.411 to Alpha, Delta, and finally the Omicron, mirroring the global trends. Time to the most recent common ancestor analyses provided estimates for the introduction of major variants, while mutation analysis revealed the widespread occurrence of D614G in the spike protein and lineage-specific mutations across structural, non-structural, and accessory proteins.Detection of the Epsilon variant (absent in other national-level studies) in November 2020, highlighted the regional heterogeneity viral spread. This study emphasizes the importance of localized genomic surveillance to capture the true diversity and evolution of SARS-CoV-2, to facilitate containment strategies in resource-limited settings.

## 1. Introduction

Since the emergence of the coronavirus disease 2019 (COVID-19), the pandemic has caused a massive disruption across the globe. Despite widespread vaccination efforts using various strategies, the causative agent, severe acute respiratory syndrome coronavirus 2 (SARS-CoV-2), has continued to spread and evolve, giving rise to variants with increased transmissibility, infectivity, and virulence, as well as decreased susceptibility to antibody neutralization compared to the wild type [1,2,3,4].

Since the identification of the first patient in March 2020, Sri Lanka experienced three major outbreaks. The first large outbreak, occurring from October 2020 to January 2021, due to the B.1.411 lineage, was completely replaced by B.1.1.7 (Alpha) in mid-April [5,6]. The outbreak due to the Alpha variant began in April 2021 and went to June 2021, with the reported number of daily cases as high as 9950, which is equivalent to around 463 cases per million population. From October 2020 to June 2021, apart from nine imported B.1.351 (Beta) and B.1.525 (Eta) cases, which were identified in overseas visitors in quarantine facilities [5,7], Sri Lanka did not experience any outbreaks due to other VOCs. Based on the reported case numbers, the largest outbreak during the study period was from July to the end of October 2021 and was predominantly due to the Delta variant and its sublineages [8]. Since October 2021, Sri Lanka experienced the tail end of Delta variant and its sublineage wave, which lasted until late 2021. However, beginning in December 2021, the Omicron variant rapidly emerged and became the dominant circulating strain, leading to a gradual increase in COVID-19 cases that peaked in January 2022. After the initial peak, case numbers declined through mid-2022. However, several Omicron sublineages including BA.2, BA.4, and BA.5 and recombinant lineages such as XXB circulated intermittently through late 2022 and into 2023. As of mid-2023, COVID-19 cases in Sri Lanka have generally stabilized with lower case counts, although regional and temporal variations continue [9,10].

These developments illustrated the challenge in controlling COVID-19, as new variants continued to shape the course of the pandemic. As such, effective control and prevention require a range of strategies. One important approach is understanding the virus’s behaviour at the regional level. Molecular characterization of SARS-CoV-2 within specific geographic regions has proven valuable for uncovering transmission dynamics, tracking viral evolution, and assessing public health impacts. Such localized genomic data help design context-specific public health interventions and the efficient use of resources.

In this context, the study focuses on the molecular epidemiology of SARS-CoV-2 infections in the Kandy District of Sri Lanka, underlining the importance of understanding the virus’s behaviour in different localities [11,12]. Located in the central region of the country, the Kandy District is shaped by various factors, such as population density, cultural practices, healthcare infrastructure, and climate, that can influence viral transmission and evolution [10,11].

Therefore, by conducting a comprehensive genomic epidemiological investigation in this region, we aimed to characterize the genetic diversity of SARS-CoV-2 across different localities within the Kandy District within the Central Province of the country, a geographically diverse region, with an estimated population of approximately 1.47 million, representing about 7% of the total national population (21,763,170), according to a 2024 census. With an area of 1940 km^2^, the district has a population density of 779 persons per Km^2^. The altitude of the area ranges from 500 m in urban areas to over 1600 m in the surrounding hill country. The region includes a mix of densely populated urban areas and isolated rural settings, supported by a diverse healthcare system with 73 primary healthcare institutions, two secondary care institutions, three tertiary care institutions, and nine special units. The diversity also provides a valuable setting for understanding local transmission dynamics and lineage distribution. This study focused on identifying circulating viral lineages, exploring potential clusters of transmission, and detecting any unique genetic signatures that could influence the local trajectory of the pandemic.

## 2. Materials and Methods

### 2.1. Selection of Study Participants

A total of 280 nasopharyngeal swabs with cycle threshold(Ct)values < 25, previously confirmed positive for SARS-CoV-2 by real-time reverse transcription PCR (rtRT-PCR), were collected between November 2020 and March 2022.Samples were selected to ensure sufficient viral load for successful sequencing and were distributed broadly throughout each month to capture temporal viral diversity. While no additional strict inclusion or exclusion criteria were applied, most samples were diagnostic samples obtained from patients attending the Out-Patient Department (OPD) with respiratory symptoms, close contacts of confirmed cases, and other clinical cases. These 280 samples represent approximately 7.4% of the total 3871 laboratoryconfirmed positive cases during the study period in the study area. Whenever possible, demographic information such as sex, age, address, travel history, and other relevant details were also collected. The study was exempted from ethical clearanceby the Ethics Review Committee of the Faculty of Medicine, University of Peradeniya (Protocol No: 2021/EC/24).

### 2.2. Nucleic Acid Extraction and SARS-CoV-2 Detection

For confirmatory SARS-CoV-2 rtRT-PCR testing, nucleic acid was extracted from nasopharyngeal swabs using SpinStar^TM^ Viral Nucleic Acid Kit 1.0 (ADT Biotech sdnbhd, Selangor, Malaysia) and Bio Flux Biospin Virus DNA/RNA Extraction Kit (Hangzhou Bioer Technology Co., Ltd., Hangzhou, China). Extractions were carried out at the Diagnostic and Research Virology Laboratory (DRVL) of the Department of Microbiology, Faculty of Medicine, University of Peradeniya.

The extracted RNA was then subjected to SARS-CoV-2 rtRT-PCR assay (Real star, Altona Diagnostics, Hamburg, Germany) on a CFX-96 real-time PCR detection system (Bio-Rad, Mississauga, ON, Canada), targeting the E and S genes, following the manufacturer’s protocol. Briefly, a total reaction volume of 30 μL was prepared using a 21.5 μL detection mixture and an 8.5 μL target RNA template. The thermal cycling conditions were as follows: reverse transcription at 45 °C for 10 min, initial denaturation at 95 °C for 3 min, followed by 45 cycles of denaturation at 95 °C for 15 s, and annealing/extension at 58 °C for 30 s. Upon completion of the assay, both positive and negative samples were stored at −80 °C for future use. A sample with a Ct value <25 was selected for whole genome sequencing (Oxford Nanopore, Oxford, UK). The sequencing was performed at DRVL.

### 2.3. Whole Genome Sequencing

SARS-CoV-2 viral RNA was re-extracted from 140 μL of stored positive samples using the QIAamp Viral RNA Mini kit (Cat No:52906, Qiagen, Hilden, Germany) following the manufacturer’s instructions. Whole genome sequencing was then performed using the SARS-CoV-2 ARTIC amplicon sequencing protocol, based on the V3 primer scheme, with the Midnight RT expansion Kit (EXP-MRT001), Rapid Barcoding Kit 96 (SQK-RBK110.96), and MinION Mk1B (Oxford Nanopore Technologies, Oxford, UK).

Briefly, complementary DNA (cDNA) synthesis was carried out using Lunascript Reverse Transcriptase SuperMix (LS RT) (New England Biolabs, Ipswich, MA, USA). Amplicons of approximately 400 base pairs were generated using the ARTIC 2019-nCoV V3 primer set through multiplex PCR. PCR products were then uniquely barcoded with the Rapid Barcoding Kit 96 (SQK-RBK110.96). Barcoded samples were then pooled and purified using AgencourtAMPure XP beads. Purified DNA was quantified using the Qubit dsDNA High-Sensitivity Assay kit (ThermoFisher Scientific, Waltham, MA, USA), and the DNA concentrations were normalized to 800 ng/μL using the elution buffer. One microlitre of Adapter (Rapid Adapter F—RAP F) was added to the final eluted barcoded DNA. Sequencing libraries were then loaded onto a MinION flow cell (R.9.4.1) for sequencing.

Consensus genome sequences, SARS-CoV-2 variant calling and mutation analysis, were performed using the EPI2ME agent workflow. All viral genome sequences generated in the study were deposited in the GISAID repository (Supplementary Table S1).

### 2.4. Phylogenetic and Mutation Analysis

For the phylogenetic analysis, all SARS-CoV-2 sequences were aligned to the reference genome, Wuhan-Hu-1 (MN908947.3), using the MAFFT FFT-NS-2 algorithm [13], excluding the 5′ and 3′ untranslated regions. A maximum likelihood tree was constructed using IQ-TREE2 version 2.3.0 with the GTR+F+I nucleotide substitution model, 1000 ultrafast bootstrap replicates (-B 1000), and the SH-aLRT branch test (-alrt 1000). The temporal signal of the sequences was assessed using TempEst v1.5.3 [14], which identified six outlier sequences that were subsequently removed. Re-analysis confirmed a strong temporal signal in the final dataset (R^2^ = 0.82; correlation coefficient = 0.91).

A time-scaled phylogenetic reconstruction was performed using BEAST v1.10.4 [15] to estimate the timing and spread of SARS-CoV-2 lineages in Sri Lanka. The analysis used the HKY substitution model with a strict molecular clock and an exponential growth coalescent tree prior. Markov Chain Monte Carlo (MCMC) sampling was run for 100 million iterations, with sampling every 1000 steps. Tracer v1.7.2 [16] was used to assess convergence, and all model parameters had effective sample sizes (ESS) above 200, ensuring adequate mixing and convergence. A maximum clade credibility (MCC) tree was generated using TreeAnnotator v1.10.4, with the first 10% of trees discarded as burn-in. The time to the most recent common ancestor (tMRCA) for each lineage was estimated and visualized using FigTree v1.4.4.

For the global phylogenetic context, we included major lineages from our dataset that were represented by more than five sequences. High-quality global SARS-CoV-2 genomes were retrieved from the RCoV19 database (https://ngdc.cncb.ac.cn/ncov, accessed on 22 January 2025). A representative sample was generated by randomly selecting one sequence per lineage per country per week during the study period (November 2020 to March 2022). Sequences were downloaded from GISAID and NCBI, resulting in a final dataset comprising 243 Sri Lankan sequences and 3363 representative global sequences from 159 countries. All sequences were aligned to the reference genome Wuhan-Hu-1 (NC_045512.2) using MAFFT FFT-NS-2, with 5′ and 3′ UTRs trimmed. A global ML phylogenetic tree was constructed in IQ-TREE2 v2.3.0 using the GTR+F+R7 model with 1000 ultrafast bootstrap replicates and SH-aLRT branch tests. Tree visualization was conducted using the ggtree package in R v4.3.2.

Amino acid substitution profiles were obtained from the GISAID data for mutation analysis. The heat map depicting the frequency and distribution of amino acid changes across different SARS-CoV-2 lineages was generated using the Complex Heatmap package (v2.16.0) in R.

## 3. Results

### 3.1. Demographic Characteristics of the Study Participants

The characteristics of the SARS-CoV-2 infected individuals (n = 280), from whom a total of 252 genomes were successfully sequenced, are summarized in Table 1. The highest proportion of the study population were symptomatic, male, and aged between 26 and 44 years.

### 3.2. Distribution of SARS-CoV-2 Lineages in the Study Area

**Figure 1:**
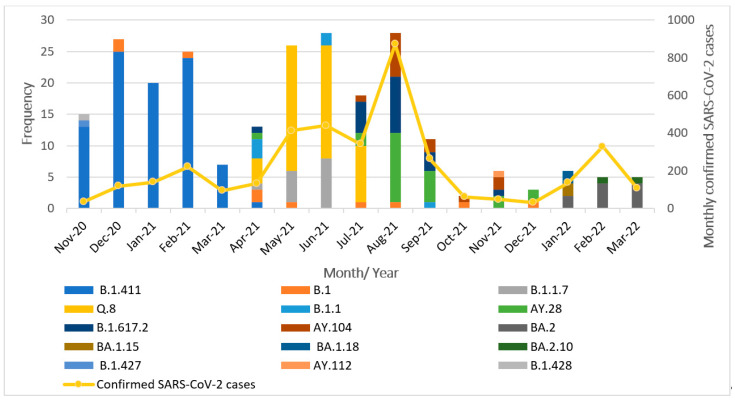
Distribution of SARS-CoV-2 lineages in the study area between November 2020 and March 2022.

The successfully sequenced 252 SARS-CoV-2 genomes were classified into various pangolin lineages. A total of 15 lineages circulated in the study area during the study period from November 2020 to March 2022. The most prevalent lineage in our dataset was B.1.411 (Sri Lankan lineage) at 36%. Among variants of concern, the Alpha variant (B.1.1.7) accounted for 5.5%, with its sublineage Q.8 accounting for 21%. The Delta variant (B.1.617.2) was present at 8%, along with its sublineages AY.28 (9.5%), AY.104 (6%), and AY.112, detected at less than 1%. The Omicron variant was represented by its sublineages BA.2 (4%), BA.1.15, BA.1.18, and BA.2.10, each present at less than 1%. The Epsilon variant (B.1.427) was also detected at a prevalence below 1%. Other lineages including B.1 (4%), B.1.1 (2%), and B.1.428 (<1%) were also detected. Overall, the B.1.411 lineage and several VOCs and their sublineages were predominantly circulating in the study area during the study period. The monthly distribution of these lineages is illustrated in Figure 1.

From November 2020 to March 2021, the B.1.411 Sri Lankan lineage was the most commonly detected variant. Notably, a genome classified as the Epsilon variant (B.1.427), a VOI, was identified in November 2020. Between April and July 2021, the Alpha variant (B.1.1.7) and its sublineage Q.8 became dominant. In July 2021, the Delta variant (B.1.617.2) and its sublineages (AY.28, AY.104) replaced Alpha and its sublineage Q.8 and became the dominant circulating strains. Delta and its sublineages continued to be the predominant variant from August to December 2021. By January 2022, the Omicron variant and its sublineages (BA.2, BA.1.15, BA.1.18, and BA.2.10) had completely replaced Delta in the study area.

These findings highlight the dynamic nature of the SARS-CoV-2 lineage distribution, with the frequency and dominance of circulating variants shifting over time.

### 3.3. Phylogenetic Analysis of Genome Sequences of SARS-CoV-2

**Figure 2:**
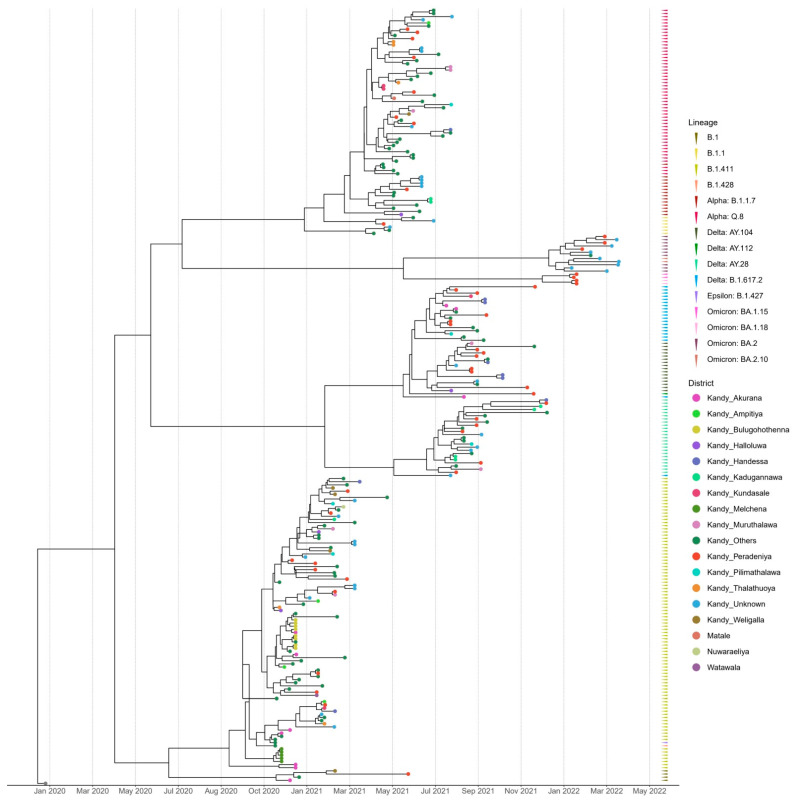
Bayesian time-scale phylogeny of SARS CoV-2 from the Kandy District. Tips are coloured by the sample location, and the external layer on the right shows the sample lineages.

The time-scaled (November 2020–March 2022) phylogenetic analysis of the SARS-CoV-2 sequences from the Kandy District of Sri Lanka revealed four distinct and well supported clades, which were grouped together according to their type of lineage (Figure 2). The sequences analyzed in this study were obtained from a range of locations across the Kandy District, which is composed of diverse urban, semi-urban, and rural settings. For instance, Akurana is a semi-urban, densely populated area with vibrant commercial activity. Melchena is a locality within the Akurana divisional secretariat and is a relatively smaller area with a semi-urban to rural land scape. Thalathuoya and Weligalla represents a more rural setting with a low population density and limited healthcare facilities. Peradeniya is a major urban centre with high population mobility and density, while Pilimathalawa lies closer to semi-urban and agricultural zones and reflects different social interactions and exposure risks.

Lineages B.1, B.1.428, and B.1.427 formed a cluster with lineage B.1.411, which was predominantly observed in the genomes sequenced from November 2020 to mid-April 2021. The genomes of the B.1.1 lineage formed a distinct cluster with the Alpha variant (B.1.1.7) and its sublineage Q.8. The Delta variant (B.1.617.2) and its sublineages AY.28, AY.104, and AY.112 formed a separate distinct cluster. Similarly, Omicron sublineages BA.1.15, BA.1.18, BA.2, and BA.2.10 formed a separate cluster that was caused by the Omicron variant (Figure 2).

**Figure 3:**
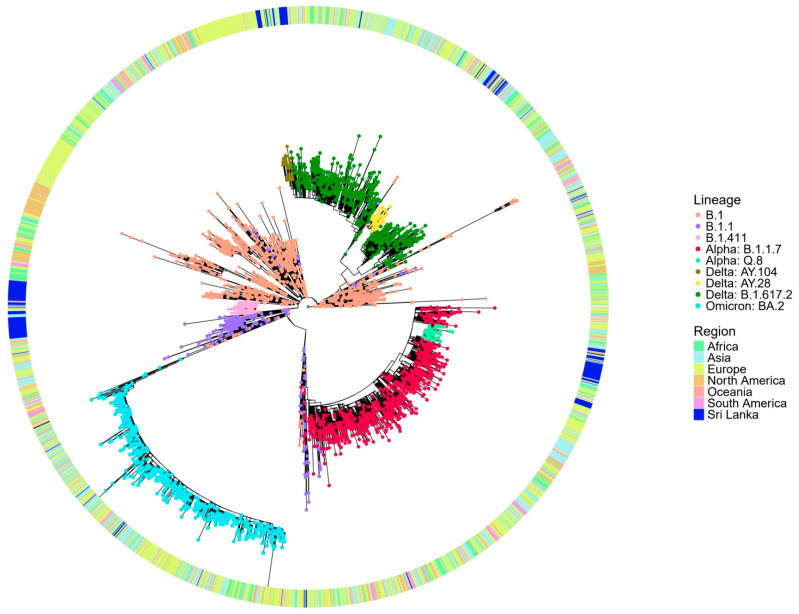
Phylogenetic analysis of Sri Lankan sequences from the study area in a global context. Maximum likelihood tree of 243 Sri Lankan sequences from the study area and 3363 representative global sequences obtained from GISAID and NCB. The tips are coloured by Sri Lankan lineages, and the external layer indicates the regions from where the representative samples are taken.

The global phylogenetic tree analysis (Figure 3) reveals that the B.1.411 lineage forms a distinct cluster composed predominantly of Sri Lankan sequences, suggesting its local circulation and independent evolution within the country. The compact clustering of the B.1.411 lineage indicates a shared common ancestor supporting the possibility of a potential founder effect and ongoing local transmission. In contrast, the B.1.1.7 (Alpha) variant appears interspersed with global sequences from multiple regions and likely represents importation and limited local transmission in the study area. The sublineages of Delta, AY.28 and AY.104 from the study area, appear more widely dispersed, but Sri Lankan sequences closely cluster within these lineages, suggesting a local transmission in the study area.

These findings suggest that the early outbreak dominated by the B.1.411 lineage was the likely result of a single or limited number of introduction events with subsequent sustained local transmission, as evidenced by its monophyletic clustering pattern. This aligns with the strict public health measures implemented in Sri Lanka during that period, including a mandatory quarantine for incoming travellers and restricted international mobility, which minimized the number of external viral introductions.

In contrast, the B.1.1.7 (Alpha)sublineages of Delta AY.28 and AY.104 appeared later when some control measures were relaxed. These lineages show a greater genetic diversity, interspersing with global sequences. This suggests multiple introductions from other countries, followed by local transmission. The easing of border restrictions and quarantine requirements may have facilitated these introductions and the subsequent spread.

The tMRCA analysis for the lineages identified in the current study suggested that the B.1.411 lineage had a tMRCA dated 12 September 2020. The 95% highest posterior density (HPD) interval for this date ranges from 12 August 2020 to 11 October 2020, indicating high confidence in this estimate. Additionally, the posterior probability (PP) of this estimate is 1, which signifies a strong statistical support for the result. The tMRCA for the Alpha (B.1.1.7) lineage in the study area is estimated to be 23 February 2021, and the 95% HPD interval for this date ranges from 3 February 2021 to 15 March 2021 with a PP of 1. The Delta variant (B.1.617.2) is estimated to have a tMRCA around 26 January 2021 with high confidence, which suggests that this ancestor existed sometime between 9 December 2020 and 11 March 2021 (95% HPD interval) with a PP of 1. The estimated tMRCA of other lineages that circulated in the study area are summarized in Table 2.

### 3.4. Patterns of Amino Acid Mutations Between Different Lineages of SARS-CoV-2 in the Study Area

The patterns of amino acid mutations between different lineages were analyzed by comparing three main types of SARS-CoV-2 proteins: structural proteins (E, M, N, and S), non-structural proteins (NSP1-NSP16), and accessory proteins (ORF3a-ORF10). The presence or absence of amino acid mutations in the lineages was displayed, highlighting the unique mutations and differences. Based on the comparison of similar lineages as determined by the phylogenetic tree, the following results were obtained.

**Figure 4:**
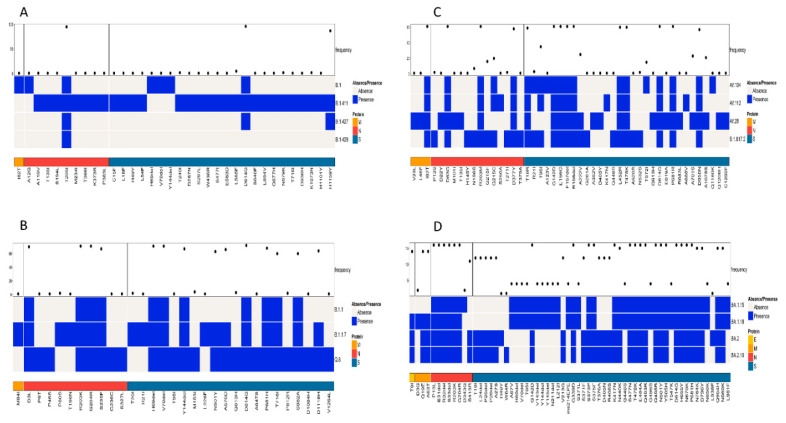
Comparison of amino acid mutations in the structural proteins of (**A**) B.1, B.1.411, B.1.427, and B.1.428 lineages, (**B**) of B.1.1, B.1.1.7, and Q.8 lineages, (**C**) Delta variant and its sublineages, and (**D**) Omicron variant and its sublineages circulated in the study area.

A simultaneous comparison of lineages B.1, B.1.411, B.1.427, and B.1.428 based on the structural proteins showed the highest frequency for D614G and H1159Y in the S protein and T205I in the N protein (Figure 4A). The structural protein regions also shared other mutations with less frequency.

Comparison of the Alpha variant (B.1.1.7) and its sublineages (Q.8 and B.1.1) based on the structural proteins showed the highest frequency for D3L, R203K, G204R, and S235F in the N protein. The highest number of amino acid mutations was observed in the S protein, with a high frequency for the following amino acid changes: H69del, V70del, D614G, Y144del, N501Y, A570D, P681H, T716I, S982A, and D1118H (Figure 4B).

A comparison of amino acid mutations in the structural proteins of the Delta variant (B.1.617.2) and its sublineages showed several mutations, with a high frequency in the M, N, and S proteins of the virus. The M protein exhibited 182T with high frequency. D63G, R203M, and D377Y were the mutations in the N protein of the virus with high frequency. The S protein exhibited the highest number of mutations. The mutations with a high frequency were D614G, P681R, G142D, E156G, F157del, R158del, L452R, T478K, D950N, and T19R (Figure 4C).

A comparison of the structural proteins of the Omicron variant and its sublineages detected in the study area showed the highest number of amino acid changes in the E, M, N, and S proteins. The S protein exhibited the highest number of mutations, followed by the N, M, and E proteins, respectively. Of the mutations, the following occurred with the highest frequency among the sequences classified as the Omicron variant: G142D, G339D, S373P, S375F, K417N, S477N, T478K, E484A, Q493R, Q4989R, N501Y, Y505H, D614G, H655Y, N679K, P681H, N440K, N764K, D796Y, Q954H, and N969K. Among the eight mutations observed in the N protein, six of them had a high frequency of occurrence. This included P13L, E31del, R32del, S33del, R203K, and G204R. The M protein of the virus had two mutations with a high frequency of occurrence (Q19E and A63T), while the E protein had a T9I mutation with a high frequency of occurrence (Figure 4D). However, the analysis of mutations in the structural proteins (E, M, N, and S) across all samples in the study identified D614G in the S protein as the most abundant mutation.

**Figure 5:**
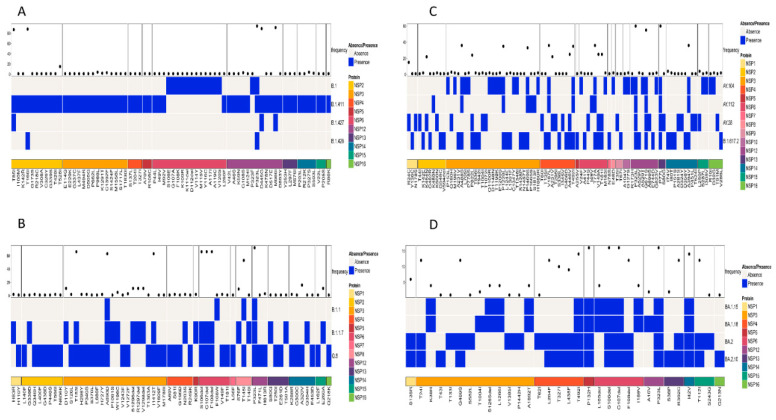
Comparison of amino acid mutations in the non-structural proteins of (**A**) B.1, B.1.411, B.1.427, and B.1.428 lineages, (**B**) of B.1.1, B.1.1.7, and Q.8 lineages, (**C**) Delta variant and its sublineages, and (**D**) Omicron variant and its sublineages circulated in the study area.

Patterns of amino acid mutation in the non-structural proteins (NSP; NSP1–NSP16) among similar lineages based on the phylogenetic tree and among all lineages were also analyzed. A comparison of mutations in the NSP of the lineages B.1, B.1.411, B.1.427, and B.1.428 identified NSP2 and NSP12 as the regions with individual mutations of high frequency. T85I and T166I were the most abundant amino acid mutations in the NSP2 region of the virus. NSP12 had three mutations occurring with a high frequency: P323L, D445G, and M666I (Figure 5A).

A comparison of amino acid mutations in the NSP of the Alpha variant and its sublineages (B.1.1.7 and Q.8) and B.1.1 revealed the NSP3, NSP6, NSP8, and NSP12 regions with high frequency mutations. The most abundant mutations in the NSP3 region were T183I, A890D, and I1412T. The NSP6 region had three deletions (S106del, G107del, and F108del) as the most abundant mutation. NSP8 (T145I) and NSP12 (P323L) had one mutation with a high frequency (Figure 5B).

Analysis of the mutations in the NSP regions of the Delta variant and its sublineages revealed that NSP12 and NSP13 were the regions with mutations of a high frequency. The most abundant mutations in the NSP12 were P323L and G671S, while P77L was the most abundant mutation in the NSP13 region of the virus. Other NSP regions of the virus also had mutations but with less frequency compared to the ones mentioned above (Figure 5C).

The Omicron variant and its sublineages had more mutations in the NSP5, NSP6, NSP12, and NSP14 regions, and relatively less mutations in other regions of the NSP protein (Figure 5D). The following mutation–NSP region pairs were identified as the most abundant mutations in the respective NSP regions of the virus: P132H—NSP5, S106del and G107del—NSP6, P323L—NSP12, and I42V—NSP14.However, the analysis of mutations across all the samples in the study identified P323L in the NSP12 region as the most frequent mutation.

**Figure 6:**
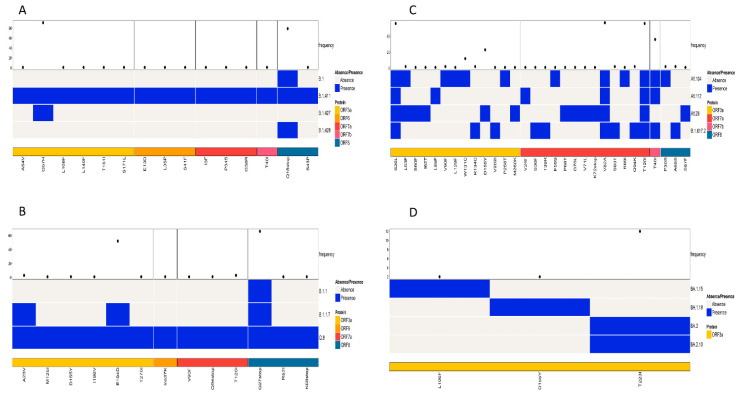
Comparison of amino acid mutations in the accessory proteins of (**A**) B.1, B.1.411, B.1.427, and B.1.428 lineages, (**B**) of B.1.1, B.1.1.7 and Q.8 lineages, (**C**) Delta variant and its sublineages, and (**D**) Omicron variant and its sublineages circulated in the study area.

The study also analyzed the patterns of amino acid mutations in the accessory proteins (ORF3a—ORF10) among similar lineages based on the phylogenetic tree and among all lineages. A comparison of mutations in the accessory proteins of the lineages B.1, B.1.411, B.1.427, and B.1.428 identified ORF3a and ORF8 as the regions containing mutations with a high frequency. The current analysis identified Q57H in the ORF3a and Q18Stop in the ORF8 regions as mutations with a high frequency (Figure 6A).

A comparison of amino acid mutations in the accessory proteins of the Alpha variant and its sublineages (B.1.1.7 and Q.8) and B.1.1 also identified ORF3a and ORF8 as the regions containing mutations with a high frequency. The predominant mutation in the ORF3a was E194D and in the ORF8 was Q27Stop (Figure 6B).

An analysis of accessory proteins of the Delta variant and its sublineages identified ORF3a, ORF7a, and ORF7b as the regions with mutations with a high frequency. The high frequency mutations identified were S26L in the ORF3a, V82A and T120I in the ORF7a, and T40I in the ORF7b regions of the virus (Figure 6C).

When the Omicron variant and its sublineages were compared for the mutations in the accessory protein regions of the virus, the study identified ORF3a as the only region with mutations with a high frequency. T223I was the most abundant mutation in the ORF3a region (Figure 6D).

## 4. Discussion

In this study, a comprehensive molecular epidemiology of SARS-CoV-2 detected from different areas of the Kandy District of Sri Lanka from November 2020 to March 2022 is presented. The first case of SARS-CoV-2 infection in Sri Lanka was reported on 27 January 2020, involving a foreign national, and the first Sri Lankan patient was identified on 10 March 2020, and a national lockdown was announced to control the spread of the virus. Over the subsequent six months (March to September 2020), the virus spread was largely contained [8]. There have been four different outbreaks reported in Sri Lanka, including the study area, and each of the four new outbreaks occurred when new variants or lineages entered during the study period. The present study classified 252 successfully sequenced genomes into 15 pangolin lineages circulating in the study area including Alpha (B.1.1.7), Delta (B.1.617.2), Omicron (B.1.1.529), and Epsilon (B.1.427), along with their sublineages. During the first five months (November 2020–March 2021) of the study, the B.1.411 lineage was the predominantly circulating lineage in the study area. A study on the genomic epidemiology of SARS-CoV-2 from different parts of the country [5] identified this lineage to be distinct from other global sequences, and their study also reports this particular lineage to be the most predominant one (231/361) circulating in the country (October 2020-April 2021). However, their study had a majority of the sequences from the Western Province with a very limited number of samples from other provinces. The present study provides comprehensive details on the epidemiology of SARS-CoV-2 in the Kandy District (Central Province), with more representative samples and covering more MOH areas in the study area. The estimated tMRCA of the B.1.411 lineage in the present study is around 12 September 2020. However, Jeewandara et al. reports an earlier tMRCA around 1 June 2020 based on the sequences they analyzed. The discrepancy could be attributed to the strict inter-provincial travel restriction in the initial stages of the pandemic, which were subsequently relaxed. These restrictions could have delayed the wider circulation and detection of the lineage across different regions. The inclusion of SARS-CoV-2 sequences derived from a wider geographic location within the Kandy District including urban, semi-urban, rural, and villages provides a comprehensive spatial perspective of the virus in the region.This also enables the identification of circulating variants across different socio-demographic and mobility contexts, reflectingthe transmission patternswith different population densities and community interactions.

Other lineages identified in the present study during the initial stages of the pandemic include B.1, B.1.1, B.1.427 (Epsilon), and B.1.428. The diversity of lineages detected in the Kandy District differed notably from those observed in the Western Province. Some variants identified in the Western Province were not detected in the present study. However, the present study identified the Epsilon variant in November 2020, which was not reported in the previous study despite samples being collected from across the country. The sequencing depth for this sample was robust, with a coverage of 172X, supporting the accuracy of the variant call and reducing the likelihood of sequencing artefact or contamination. Nevertheless, given that only one Epsilon variant sequence was detected among a limited number of positive samples analyzed, we cannot exclude the possibility that this represents an isolated imported case rather than sustained local transmission. Consequently, the contribution of the Epsilon variant to the initial surge in COVID-19 cases in Sri Lanka remains uncertain. Detailed epidemiological investigations, including re-sequencing, phylogenetic analyses, and the integration of travel and contact history data, would be required to definitively determine the significance of this detection. Furthermore, due to the limited sample size and sequencing scope, we acknowledge that an accurate estimation of the true prevalence of circulating lineages during this period is not possible.

The shift in dominant lineages in the study area over time, from B.1.411 (November 2020 to March 2021) to Alpha (B.1.1.7) between April and July 2021, to Delta (B.1.617.2) from August to December 2021, and finally to Omicron (B.1.1.529) from January 2022 onwards, closely mirrors global trends, where more transmissible variants progressively displaced their predecessors.

The tMRCA analysis provided insights into the timing of introductions and the spread of different lineages in the study area. The Alpha variant had a tMRCA of 23 February 2021, aligning with its global emergence and spread in early 2021. The Delta variant’s tMRCA of 26 January 2021 falls within the period when it began to rapidly replace other variants globally. These dates provide a temporal framework for understanding the introduction and spread of these lineages in Sri Lanka, particularly in the Kandy District.

The detailed analysis of amino acid mutations across structural, non-structural, and accessory proteins provides valuable insights into the SARS-CoV-2 evolutionary dynamics. The D614G mutation in the S protein was the most abundant mutation observed across all samples. This mutation has been widely reported globally and currently occurs in all VOCs, suggesting its central role. Studies have investigated the effect of this mutation, and the findings show an enhanced association with increased infectivity and transmissibility in different cell lines and species [3,4,17].

A structural protein analysis showed lineage-specific mutation patterns. For instance, the B.1.411 lineage and its related lineages (B.1, B.1.427, and B.1.428) exhibited high frequencies of D614G and H1159Y in the S protein and T205I in the N protein. The Alpha variant (B.1.1.7) and its sublineages showed characteristic mutations such as H69del, V70del, and N501Y in the S protein, which are associated with increased transmissibility and immune evasion [18]. The Delta variant exhibited multiple high-frequency mutations in the S protein (e.g., L452R, T478K) and in the N protein (e.g., R203M, D377Y), which contribute to its increased fitness and immune evasion [19,20]. Omicron and its sublineages showed the highest number of mutations in the S protein, such as G339D, S373P, and N501Y, which are linked to significant changes in the antigenic properties of the virus [21].

An analysis of the NSP regions identified P323L in NSP12 as the most abundant mutation across all samples, a mutation that is associated with enhanced viral replication fidelity [22]. Specific lineages showed distinct NSP mutation patterns: T85I and T166I in NSP2 for B.1.411-related lineages and P132H in NSP5 for Omicron lineages. Studies suggest that these mutations may contribute to their respective replication efficiencies and pathogenicity [23].

An accessory protein analysis highlighted mutations in ORF3a and ORF8 as significant across multiple lineages. Mutations like Q57H in ORF3a and Q18Stop in ORF8 for B.1.411-related lineages and S26L in ORF3a for the Delta variant may impact the virus’s ability to evade host immune responses and thus modulate its pathogenesis [24,25,26].

The temporal shift in the frequency of the mutations aligns with the epidemiological progression of SARS-CoV-2 in the study area. The increasing prevalence of the mutation H1159Y in the B.1.411 lineage during the early pandemic phases in the region suggest a possible local adaptation or selective advantage. While the direct functional effects of H1159Y remains to be studied, its stable prevalence supports the hypothesis of lineage fitness that warrants further investigation. Similarly, the Alpha variant and its sub-lineages are characterized by several key mutations which have been strongly linked to an increased viral transmissibility and potential changes in pathogenicity. Some of these mutations facilitate more efficient viral entry into the host cell and contributed to the rapid spread globally and in Sri Lanka during early 2021. In the context of Sri Lanka, the presence of Alpha and its sublineages such as Q.8 marked a significant phase in local epidemics, contributing to increased case numbers and requiring an adjustment in public health responses.

The rise in spike protein mutations associated with the Delta variant and its sublineages coincides with a significant increase in SARS-CoV-2 infections locally during mid to late 2021, reflecting the enhanced transmissibility reported globally. Similarly, the emergence of Omicron-associated mutations paralleled a rapid increase in cases in late 2021, aligning with a rapid spike in case numbers locally [8,27].

The findings of the current study were not only consistent with global trends in the evolution and spread of SARS-CoV-2 but also highlight unique local dynamics in the Kandy District of Sri Lanka. The early dominance of the B.1.411 lineage and the subsequent shifts to the Alpha, Delta, and Omicron variants reflected the complex interplay of viral evolution, human behaviour, and public health interventions.

## 5. Limitations of the Study

The study has several limitations that should be considered when interpreting the results. Firstly, the sampling strategy was non-systematic and based on available positive samples with an adequate viral load rather than a randomized or population-representative design. Consequently, the genomic data may not fully capture the true diversity or prevalence of SARS-CoV-2 lineages circulating in the study area during the investigation period. Secondly, detailed epidemiological data on overall monthly COVID-19 case counts in the Kandy District were not integrated into the analysis, limiting our ability to assess sequencing coverage relative to the epidemic’s temporal dynamics. This constrains quantitative inference about the representativeness of the sequenced samples in relation to the total burden of infections. Third, the exclusion criteria for sample selection were not explicit, raising the potential for the biased inclusion of cases, such as the preferential sequencing of samples with higher viral loads or from particular geographic or clinical sub-populations. Such biases could influence observed lineage frequencies and mutation patterns. Additionally, the limited sample size and sequencing scope restrict the generalizability of the results and may have hindered the identification of low-prevalence lineages or emerging mutations. This is exemplified by the single detection of the Epsilon variant, which could represent an isolated importation event rather than sustained community transmission but could not be definitively characterized due to limited data. Fourth, in phylogenetic analyses, the global dataset used for comparison included one representative sequence per lineage per country per week to ensure broad temporal and geographic coverage. However, this sparse sampling outside of Sri Lanka limits the detection of country-specific clustering or regional evolutionary patterns, restricting comprehensive international comparisons and possibly underestimating transmission linkages. Finally, the study did not include functional assays or detailed clinical and contact-tracing data that would enable the direct assessment of the impact of identified mutations on viral transmissibility, immune escape, or disease severity. Despite these limitations, our findings provide valuable regional insights into the evolution and dynamics of SARS-CoV-2 lineages and mutations in Sri Lanka.

## 6. Conclusions

This study presented a comprehensive molecular epidemiological analysis of SARS-CoV-2 detected in the Kandy District of Sri Lanka from November 2020 to March 2022. The findings highlight the dynamic shifts in circulating viral lineages, from the early dominance of the locally emerged B.1.411 lineage to successive waves of the Alpha, Delta, and Omicron variants, mirroring the global trends. The identification of multiple mutations across structural, non-structural, and accessory proteins provides descriptive information on the genetic diversity of the circulating SARS-CoV-2 lineages in the study area.The mutation patterns warrant further investigation to understand their potential roles in viral adaptation, transmissibility, and immune evasion. Notably, the detection of the Epsilon variant, absent in prior national studies, underscored the importance of geographically representative surveillance. These findings emphasize the need for continuous genomic surveillance to monitor viral evolutionto inform public health responses, and guide containment strategies in future outbreaks.

## Figures and Tables

**Table 1:** Demographic characteristics of the study participants.

Variables	Descriptive Statistics (n = 280)
Age (years), mean±SD	37.21 ± 20.13
Age group (years), n(%)	
1–13	31 (11%)
14–25	56 (20%)
26–44	92 (32.9%)
45–60	62 (22.1%)
>60	39 (13.9%)
Gender, n	
Male	168 (60%)
Female	112 (40%)
Clinical profile, n	
Asymptomatic	85 (30.4%)
Symptomatic	195 (69.6%)

**Table 2:** Computed tMRCA and HPD intervals for each identified lineages in the study area.

Lineage	tMRCA	tMRCA Date	95% Highest Posterior Density (HPD)	95% Highest Posterior Density Date (HPD)	Posterior Probability (PP)
B.1.411	2020.6976	12 September 2020	2020.6123–2020.7767	12 August 2020–11 October 2020	1
B.1	2020.8803	18 November 2020	2020.7812–2020.9344	12 October 2020–7 December 2020	0.9364
B.1.1	2020.9903	28 December 2020	2020.8733–2021.096	15 November 2020–5 February 2021	1
B.1.617.2	2021.0698	26 January 2021	2020.9386–2021.1892	9 December 2020–11 March 2021	1
B.1.1.7	2021.1462	23 February 2021	2021.0915–2021.2003	3 February 2021–15 March 2021	1
Q.8	2021.2233	24 March 2021	2021.1862–2021.2591	10 March 2021–6 April 2021	1
AY.28	2021.4659	20 June 2021	2021.413–2021.515	1 June 2021–8 July 2021	1
AY.104	2021.4677	21 June 2021	2021.421–2021.5131	4 June 2021–7 July 2021	1
BA.2	2021.9412	11 December 2021	2021.8824–2021.9967	19 November 2021–31 December 2021	1
BA.1.15	2022.0224	9 January 2022	2021.9911–2022.0384	29 December 2021–15 January 2022	1
BA.1.18	2022.0308	12 January 2022	2021.9951–2022.0493	30 December 2021–19 January 2022	1

## Data Availability

The original data presented in the study are openly available in GISAID (see Supplementary Table S1).

## Supplementary Materials

The following supporting information can be downloaded at: https://www.mdpi.com/article/10.3390/v17091189/s1, Table S1: Details of the SARS-CoV-2 sequences submitted to GISAID.
