# Supplementary material for: Molecular Epidemiology of SARS-CoV-2 Detected from Different Areas of the Kandy District of Sri Lanka from November 2020–March 2022
Source: Viruses. 2025 Aug 29;17(9):1189. doi: 10.3390/v17091189 (PMC12474409; doi:10.3390/v17091189)
Supplement: Supplementary file 1 [file viruses-17-01189-s001.zip › viruses-3786314-supplementary.pdf]

Table S1. Details of SARS-CoV-2 sequences submitted to GISAID from the Diagnostic and Research Virology Laboratory (DRVL), Department of Microbiology, Faculty of Medicine, University of Peradeniya, Sri Lanka.

Accession ID	Location	Gender	Age of the Patient
EPI_ISL_1781176 3	Asia / Sri Lanka / Kandy / Udaperadeniya	Female	54
EPI_ISL_1781265 4	Asia / Sri Lanka / Matale	Male	38
EPI_ISL_1781267 1	Asia / Sri Lanka / Kandy / Handessa / Udunuwara	Male	35
EPI_ISL_1781273 7	Asia / Sri Lanka / Kandy	Male	Nor known
EPI_ISL_1785936 4	Asia / Sri Lanka / Kandy / Peradeniya / Gangawatakorala / University Park	Female	73
EPI_ISL_1785936 6	Asia / Sri Lanka / Kandy / Kiribathkumbura / Yatinuwara	Male	17
EPI_ISL_1785936 7	Asia / Sri Lanka / Kandy / Muruthalawa / Yatinuwara	Male	65
EPI_ISL_1785936 8	Asia / Sri Lanka / Kandy / Peradeniya / Kiribathkumbura / Yatinuwara	Female	39
EPI_ISL_1785936 9	Asia / Sri Lanka / Kandy / Peradeniya / Udaperadeniya	Male	64
EPI_ISL_1785937 0	Asia / Sri Lanka / Kandy / Peradeniya / Muruthalawa	Male	51
EPI_ISL_1785937 1	Asia / Sri Lanka / Kandy / Nawalapitiya	Female	32
EPI_ISL_1795758 5	Asia / Sri Lanka / Kandy / Ayoganagama / Pallekele / Kundasale	Male	63
EPI_ISL_1795758 6	Asia / Sri Lanka / Kandy / Bokkawala	Male	50
EPI_ISL_1795763 2	Asia / Sri Lanka / Kandy	Male	Not known
EPI_ISL_1795763 8	Asia / Sri Lanka / Kandy / Hnadessa / Udunuwara	Female	18
EPI_ISL_1797303 0	Asia / Sri Lanka / Kandy / Melchena / Akurana	Male	6
EPI_ISL_1797303 1	Asia / Sri Lanka / Kandy / Pitawalawatta / Gelioya	Male	44
EPI_ISL_1797303 2	Asia / Sri Lanka / Kandy / Dodangolla / Akurana	Female	33
EPI_ISL_1797303 3	Asia / Sri Lanka / Kandy / Dodangolla / Akurana	Male	55
EPI_ISL_1797303 4	Asia / Sri Lanka / Kandy / Bulugohatenna / Akurana	Male	18

EPI_ISL_17973035	Asia / Sri Lanka / Kandy / Bulugohatenna / Akurana	Male	13
EPI_ISL_17973036	Asia / Sri Lanka / Kandy / Bulugohatenna / Akurana	Male	8
EPI_ISL_17973037	Asia / Sri Lanka / Kandy / Malpana / Kengalla / Menikhinna	Male	3
EPI_ISL_18105318	Asia / Sri Lanka / Kandy	Male	2
EPI_ISL_18106309	Asia / Sri Lanka / Kandy / Peradeniya	Male	33
EPI_ISL_18106385	Asia / Sri Lanka / Kandy / Peradeniya / Doluwa	Male	24
EPI_ISL_18106386	Asia / Sri Lanka / Kandy	Female	49
EPI_ISL_18108258	Asia / Sri Lanka / Kandy / Bogambara	Male	55
EPI_ISL_18108259	Asia / Sri Lanka / Kandy / Weliwita	Female	48
EPI_ISL_18108284	Asia / Sri Lanka / Kandy / Thalathuoya	Male	46
EPI_ISL_18108901	Asia / Sri Lanka / Kandy / Bogambara	Male	48
EPI_ISL_18108902	Asia / Sri Lanka / Kandy / Peradeniya / Gelioya	Female	67
EPI_ISL_18108903	Asia / Sri Lanka / Kandy	Male	54
EPI_ISL_18108905	Asia / Sri Lanka / Kandy / Poojapitiya	Male	33
EPI_ISL_18108907	Asia / Sri Lanka / Kandy / Gelioya	Female	42
EPI_ISL_18108910	Asia / Sri Lanka / Kandy / Pilimathalawa	Male	43
EPI_ISL_18108913	Asia / Sri Lanka / Kandy / Weligalla	Female	19
EPI_ISL_18108956	Asia / Sri Lanka / Kandy / Kadugannawa	Male	29
EPI_ISL_18108957	Asia / Sri Lanka / Kandy / Katugasthota	Male	56
EPI_ISL_18108984	Asia / Sri Lanka / Kandy / Halloluwa	Male	41
EPI_ISL_18109033	Asia / Sri Lanka / Kandy / Kulugammana / Harispattuwa	Female	9
EPI_ISL_18109034	Asia / Sri Lanka / Kandy / Loolwatta / Ududumbara	Female	27
EPI_ISL_18109035	Asia / Sri Lanka / Kandy / Peradeniya	Male	21
EPI_ISL_1810903	Asia / Sri Lanka / Kandy	Female	33

6			
EPI_ISL_18109037	Asia / Sri Lanka / Kandy / Gelioya	Female	21
EPI_ISL_18111765	Asia / Sri Lanka / Kandy / Peradeniya / Hindagala / Gangawatakorala	Male	36
EPI_ISL_18111766	Asia / Sri Lanka / Kandy / Kadugannawa	Female	26
EPI_ISL_18111767	Asia / Sri Lanka / Kandy / Werellagama / Harispattuwa	Female	31
EPI_ISL_18111768	Asia / Sri Lanka / Kandy	Male	15
EPI_ISL_18111769	Asia / Sri Lanka / Kandy	Male	65
EPI_ISL_18111773	Asia / Sri Lanka / Kandy	Female	60
EPI_ISL_18111791	Asia / Sri Lanka / Kandy / Katugasthota / Harispattuwa	Male	26
EPI_ISL_18111794	Asia / Sri Lanka / Kandy / Balagolla / Kengalla	Female	22
EPI_ISL_18111796	Asia / Sri Lanka / Kandy / Menikdiwela	Female	52
EPI_ISL_18111797	Asia / Sri Lanka / Kandy / Thawalanthenna / Panvila	Female	51
EPI_ISL_18111798	Asia / Sri Lanka / Kandy / Deltota / Pattiyagama	Male	23
EPI_ISL_18111801	Asia / Sri Lanka / Kandy / Thalathuoya	Male	58
EPI_ISL_18111802	Asia / Sri Lanka / Kandy / Sangarajapura / Hataraliyadda	Male	55
EPI_ISL_18111829	Asia / Sri Lanka / Kandy / Katugasthota / Harispattuwa	Male	58
EPI_ISL_18111873	Asia / Sri Lanka / Kandy / Peradeniya	Female	46
EPI_ISL_18112009	Asia / Sri Lanka / Kandy	Male	Not known
EPI_ISL_18112010	Asia / Sri Lanka / Kandy / Peradeniya / Gelioya	Male	54
EPI_ISL_18112120	Asia / Sri Lanka / Kandy / Peradeniya	Male	22
EPI_ISL_18118615	Asia / Sri Lanka / Kandy / Ampitiya	Female	28
EPI_ISL_18118617	Asia / Sri Lanka / Kandy / Poojapitiya	Male	29
EPI_ISL_18118619	Asia / Sri Lanka / Kandy / Kiribathkubura	Female	45
EPI_ISL_18118654	Asia / Sri Lanka / Kandy / Bulugohotenna / Akurana	Male	56

EPI_ISL_1811865<br>6	Asia / Sri Lanka / Kandy / Bulugohatenna / Akurana	Male	21
EPI_ISL_1811870<br>9	Asia / Sri Lanka / Kandy / Bulugohotenna / Akurana	Female	16
EPI_ISL_1811872<br>0	Asia / Sri Lanka / Kandy / Bulugohotenna / Akurana	Female	20
EPI_ISL_1811873<br>4	Asia / Sri Lanka / Kandy / Bulugohotenna / Akurana	Female	25
EPI_ISL_1811873<br>6	Asia / Sri Lanka / Kandy / Gampola	Female	25
EPI_ISL_1811873<br>7	Asia / Sri Lanka / Kandy / Peradeniya / Gangawatakorale	Female	73
EPI_ISL_1811873<br>8	Asia / Sri Lanka / Kandy / Kiribathkumbura	Male	17
EPI_ISL_1811873<br>9	Asia / Sri Lanka / Kandy / Muruthalawa	Male	65
EPI_ISL_1811874<br>1	Asia / Sri Lanka / Kandy / Peradeniya / Yatinuwara	Female	39
EPI_ISL_1811874<br>2	Asia / Sri Lanka / Kandy / Muruthalawa	Male	51
EPI_ISL_1811877<br>2	Asia / Sri Lanka / Kandy / Handessa / Udunuwara	Male	35
EPI_ISL_1811880<br>1	Asia / Sri Lanka / Kandy / Handessa / Udunuwara	Female	18
EPI_ISL_1811882<br>1	Asia / Sri Lanka / Kandy / Udaperadeniya	Female	54
EPI_ISL_1823327<br>2	Asia / Sri Lanka / Kandy / Dodangolla / Akurana	Male	30
EPI_ISL_1823327<br>4	Asia / Sri Lanka / Kandy / Pangollamada / Akurana	Male	26
EPI_ISL_1823327<br>6	Asia / Sri Lanka / Kandy / Mawilmada	Female	8
EPI_ISL_1823327<br>9	Asia / Sri Lanka / Kandy	Male	7
EPI_ISL_1823328<br>1	Asia / Sri Lanka / Kandy / Yatinuwara	Female	32
EPI_ISL_1823328<br>5	Asia / Sri Lanka / Kandy / Egodawatte	Female	74
EPI_ISL_1823328<br>7	Asia / Sri Lanka / Kandy / Peradeniya / Udaperadeniya	Female	51
EPI_ISL_1823328<br>9	Asia / Sri Lanka / Kandy / Galagedara	Female	52
EPI_ISL_1823329<br>2	Asia / Sri Lanka / Kandy / Sudhumpola	Male	14
EPI_ISL_1823329<br>4	Asia / Sri Lanka / Kandy / Yatihalagala / Harispattuwa	Male	58
EPI_ISL_1823329	Asia / Sri Lanka / Kandy	Male	Not known

6			
EPI_ISL_18233299	Asia / Sri Lanka / Kandy / Danthure	Male	45
EPI_ISL_18233301	Asia / Sri Lanka / Kandy	Male	61
EPI_ISL_18233304	Asia / Sri Lanka / Kandy / Mahayyawa	Male	36
EPI_ISL_18233305	Asia / Sri Lanka / Kandy / Leemagahakotuwa / Akurana	Male	3
EPI_ISL_18233307	Asia / Sri Lanka / Kandy	Male	55
EPI_ISL_18233403	Asia / Sri Lanka / Kandy / Akurana	Male	68
EPI_ISL_18233404	Asia / Sri Lanka / Kandy / Melchena / Akurana	Female	72
EPI_ISL_18233406	Asia / Sri Lanka / Kandy / Haragama / Gurudeniya	Female	7
EPI_ISL_18233409	Asia / Sri Lanka / Kandy / Akurana	Female	52
EPI_ISL_18233491	Asia / Sri Lanka / Kandy / Handessa	Male	35
EPI_ISL_18233534	Asia / Sri Lanka / Kandy	Female	23
EPI_ISL_18233537	Asia / Sri Lanka / Kandy	Male	18
EPI_ISL_18233539	Asia / Sri Lanka / Nuwaraeliya	Male	Not known
EPI_ISL_18233540	Asia / Sri Lanka / Kandy	Female	2
EPI_ISL_18233541	Asia / Sri Lanka / Kandy / Murutalawa / Yatinuwara	Male	46
EPI_ISL_18233567	Asia / Sri Lanka / Kandy / Muruthalawa / Yatinuwara	Female	38
EPI_ISL_18233607	Asia / Sri Lanka / Kandy / Kadugannawa	Female	53
EPI_ISL_18233609	Asia / Sri Lanka / Kandy / Pilapitiya / Yatinuwara	Male	67
EPI_ISL_18233610	Asia / Sri Lanka / Kandy / Peradeniya	Female	56
EPI_ISL_18233611	Asia / Sri Lanka / Kandy / Kadugannawa / Yatinuwara	Female	25
EPI_ISL_18233612	Asia / Sri Lanka / Kandy / Pilimathalawa / Yatinuwara	Male	22
EPI_ISL_18233657	Asia / Sri Lanka / Kandy / Danthure	Male	7
EPI_ISL_18233658	Asia / Sri Lanka / Kandy / Kolabissa / Galaha	Male	Not known

EPI_ISL_18233878	Asia / Sri Lanka / Kandy / Pallegama / Menikhinna	Female	Not known
EPI_ISL_18233879	Asia / Sri Lanka / Kandy / Pasbage / Nawalapitiya	Female	16
EPI_ISL_18233880	Asia / Sri Lanka / Kandy / Panvila	Male	3
EPI_ISL_18233881	Asia / Sri Lanka / Kandy / Ampitiya	Male	54
EPI_ISL_18233882	Asia / Sri Lanka / Kandy / Menikdiwela	Female	26
EPI_ISL_18233883	Asia / Sri Lanka / Kandy / Kundasale	Female	97
EPI_ISL_18233884	Asia / Sri Lanka / Kandy / Galhinna	Male	1
EPI_ISL_18385165	Asia / Sri Lanka / Kandy	Male	Not known
EPI_ISL_18385251	Asia / Sri Lanka / Kandy / Koshinna	Female	29
EPI_ISL_18385286	Asia / Sri Lanka / Kandy / Polgolla / Wattegama	Female	22
EPI_ISL_18385287	Asia / Sri Lanka / Kandy / Ampitiya	Male	3
EPI_ISL_18385373	Asia / Sri Lanka / Kandy / Gangawata Korale	Male	66
EPI_ISL_18385375	Asia / Sri Lanka / Kandy / Gangawata Korale	Male	27
EPI_ISL_18385378	Asia / Sri Lanka / Kandy / Thalathuoya	Female	44
EPI_ISL_18385380	Asia / Sri Lanka / Kandy / Alawathugoda / Akurana	Male	3
EPI_ISL_18385386	Asia / Sri Lanka / Kandy / Thalathuoya	Female	55
EPI_ISL_18385419	Asia / Sri Lanka / Kandy / Kadugannawa	Female	44
EPI_ISL_18385421	Asia / Sri Lanka / Kandy / Melchena / Akurana	Male	36
EPI_ISL_18390057	Asia / Sri Lanka / Kandy / Melchena / Akurana	Female	36
EPI_ISL_18390058	Asia / Sri Lanka / Kandy / Melchena / Akurana	Female	8
EPI_ISL_18390810	Asia / Sri Lanka / Kandy / Batugoda	Male	68
EPI_ISL_18390811	Asia / Sri Lanka / Kandy / Thalathuoya	Male	14
EPI_ISL_18390812	Asia / Sri Lanka / Kandy / Muruthalawa	Male	51
EPI_ISL_1839085	Asia / Sri Lanka / Kandy / Welamboda / Udunuwara	Male	46

2			
EPI_ISL_18390853	Asia / Sri Lanka / Kandy	Female	28
EPI_ISL_18390854	Asia / Sri Lanka / Kandy / Madulkele / Panvila	Male	36
EPI_ISL_18390873	Asia / Sri Lanka / Kandy / Pilimathalawa	Female	16
EPI_ISL_18390915	Asia / Sri Lanka / Kandy / Heerassagala / Gangawata Korale / University Park	Male	37
EPI_ISL_18390916	Asia / Sri Lanka / Kandy / Batagalla / Poojapitiya	Male	19
EPI_ISL_18390917	Asia / Sri Lanka / Kandy / Peradeniya	Female	52
EPI_ISL_18390918	Asia / Sri Lanka / Kandy / Akurana	Male	8
EPI_ISL_18390919	Asia / Sri Lanka / Kandy / Galaha	Male	18
EPI_ISL_18390920	Asia / Sri Lanka / Kandy	Male	76
EPI_ISL_18390921	Asia / Sri Lanka / Kandy / Udunuwara	Male	15
EPI_ISL_18390922	Asia / Sri Lanka / Kandy / Doluwa	Male	26
EPI_ISL_18390923	Asia / Sri Lanka / Kandy / Hasalaka	Male	1
EPI_ISL_18390924	Asia / Sri Lanka / Kandy / Akurana	Male	24
EPI_ISL_18390925	Asia / Sri Lanka / Kandy / Akurana	Female	90
EPI_ISL_18390926	Asia / Sri Lanka / Kandy / Bambaradeniya	Male	27
EPI_ISL_18390927	Asia / Sri Lanka / Kandy / Peradeniya	Male	Not known
EPI_ISL_18390928	Asia / Sri Lanka / Kandy / Peradeniya	Male	Not known
EPI_ISL_18390929	Asia / Sri Lanka / Kandy	Male	33
EPI_ISL_18390930	Asia / Sri Lanka / Kandy / Pilimathalawa / Udunuwara	Male	25
EPI_ISL_18390931	Asia / Sri Lanka / Kandy / Hnadessa / Udunuwara	Male	2
EPI_ISL_18390932	Asia / Sri Lanka / Kandy / Akurana	Female	55
EPI_ISL_18454485	Asia / Sri Lanka / Kandy / Welamboda	Male	29
EPI_ISL_18454528	Asia / Sri Lanka / Kandy / Aniwatta	Male	30

EPI_ISL_18454529	Asia / Sri Lanka / Kandy / Peradeniya / Udaperadeniya	Female	60
EPI_ISL_18454530	Asia / Sri Lanka / Kandy	Female	16
EPI_ISL_18454531	Asia / Sri Lanka / Kandy / Kundasale	Male	61
EPI_ISL_18454532	Asia / Sri Lanka / Kandy / Kundasale	Female	53
EPI_ISL_18454533	Asia / Sri Lanka / Kandy / Handessa	Male	27
EPI_ISL_18454535	Asia / Sri Lanka / Kandy	Male	44
EPI_ISL_18454552	Asia / Sri Lanka / Kandy	Female	37
EPI_ISL_18454553	Asia / Sri Lanka / Kandy	Female	29
EPI_ISL_18454853	Asia / Sri Lanka / Kandy / Peradeniya	Female	21
EPI_ISL_18454854	Asia / Sri Lanka / Kandy / Peradeniya	Female	22
EPI_ISL_18454856	Asia / Sri Lanka / Kandy / Pangollamada / Akurana	Male	Not known
EPI_ISL_18454861	Asia / Sri Lanka / Kandy / Gangawata Korale	Female	29
EPI_ISL_18454865	Asia / Sri Lanka / Kandy / Akurana	Male	71
EPI_ISL_18454868	Asia / Sri Lanka / Kandy / Peradeniya	Male	62
EPI_ISL_18454869	Asia / Sri Lanka / Kandy / Penideniya	Male	1
EPI_ISL_18460506	Asia / Sri Lanka / Kandy	Male	57
EPI_ISL_18460507	Asia / Sri Lanka / Kandy / Kahawatta / Ambatenna	Male	33
EPI_ISL_18460508	Asia / Sri Lanka / Kandy / Penideniya	Male	32
EPI_ISL_18460509	Asia / Sri Lanka / Kandy / Halloluwa	Male	45
EPI_ISL_18460510	Asia / Sri Lanka / Kandy / Kundasale	Female	66
EPI_ISL_18460511	Asia / Sri Lanka / Kandy / Peradeniya	Male	20
EPI_ISL_18460512	Asia / Sri Lanka / Kandy	unknown	83
EPI_ISL_18460513	Asia / Sri Lanka / Kandy / Handessa	Male	44
EPI_ISL_1846051	Asia / Sri Lanka / Kandy / Weligalla	Male	28

4			
EPI_ISL_1846051 5	Asia / Sri Lanka / Kandy / Dunuwila	Female	35
EPI_ISL_1846070 0	Asia / Sri Lanka / Kandy / Kadugannawa	Male	51
EPI_ISL_1846120 1	Asia / Sri Lanka / Kandy / Halloluwa	Male	28
EPI_ISL_1846129 2	Asia / Sri Lanka / Kandy / Ampitiya	Male	65
EPI_ISL_1846137 0	Asia / Sri Lanka / Kandy / Dunuwila	Male	5
EPI_ISL_1846147 7	Asia / Sri Lanka / Kandy	Male	Not known
EPI_ISL_1846147 8	Asia / Sri Lanka / Watawala	Male	1
EPI_ISL_1846147 9	Asia / Sri Lanka / Kandy / Peradeniya	Male	34
EPI_ISL_1846148 0	Asia / Sri Lanka / Kandy / Doragamuwa	Female	20
EPI_ISL_1846148 1	Asia / Sri Lanka / Kandy / Hanthana	Female	25
EPI_ISL_1846148 2	Asia / Sri Lanka / Kandy / Weligalla	Female	61
EPI_ISL_1846150 1	Asia / Sri Lanka / Kandy / Weligalla	Male	53
EPI_ISL_1846243 8	Asia / Sri Lanka / Kandy / Pilimathalawa	Male	39
EPI_ISL_1846243 9	Asia / Sri Lanka / Kandy / Akurana	Male	3
EPI_ISL_1846244 0	Asia / Sri Lanka / Kandy / Akurana	Female	38
EPI_ISL_1846244 1	Asia / Sri Lanka / Kandy / Akurana	Female	8
EPI_ISL_1846244 2	Asia / Sri Lanka / Kandy / Peradeniya	Male	49
EPI_ISL_1846244 3	Asia / Sri Lanka / Kandy / Peradeniya / Gelioya	Male	17
EPI_ISL_1846244 4	Asia / Sri Lanka / Kandy / Pallegama	Male	38
EPI_ISL_1846244 5	Asia / Sri Lanka / Kandy / Peradeniya / Gelioya	Female	24
EPI_ISL_1846248 4	Asia / Sri Lanka / Kandy / Udispaththuwa	Female	36
EPI_ISL_1846248 5	Asia / Sri Lanka / Kandy / Peradeniya	Female	24
EPI_ISL_1847243 5	Asia / Sri Lanka / Kandy / Pallekele	Male	54

EPI_ISL_18472436	Asia / Sri Lanka / Kandy / Peradeniya	Female	12
EPI_ISL_18472437	Asia / Sri Lanka / Kandy / Udailuka	Female	26
EPI_ISL_18472438	Asia / Sri Lanka / Kandy / Galagedara	Female	28
EPI_ISL_18472439	Asia / Sri Lanka / Kandy / Menikdiwela	Male	58
EPI_ISL_18472440	Asia / Sri Lanka / Kandy / Halloluwa	Male	40
EPI_ISL_18472719	Asia / Sri Lanka / Kandy / Peradeniya / Doluwa	Male	23
EPI_ISL_18472720	Asia / Sri Lanka / Kandy / Peradeniya	Female	26
EPI_ISL_18472721	Asia / Sri Lanka / Kandy / Hondiyadeniya	Male	32
EPI_ISL_18472722	Asia / Sri Lanka / Kandy / Metiwalatenna	Male	28
EPI_ISL_18472723	Asia / Sri Lanka / Kandy / Handessa	Female	61
EPI_ISL_18472724	Asia / Sri Lanka / Kandy	Female	24
EPI_ISL_18472725	Asia / Sri Lanka / Kandy / Peradeniya	Female	25
EPI_ISL_18472726	Asia / Sri Lanka / Kandy / Peradeniya	Female	21
EPI_ISL_18472727	Asia / Sri Lanka / Kandy	Male	Not known
EPI_ISL_18472728	Asia / Sri Lanka / Kandy / Peradeniya	Male	25
EPI_ISL_18472769	Asia / Sri Lanka / Kandy / Weligalla	Male	49
EPI_ISL_18472771	Asia / Sri Lanka / Kandy	Female	40
EPI_ISL_18472772	Asia / Sri Lanka / Kandy / Peradeniya	Female	46
EPI_ISL_18472773	Asia / Sri Lanka / Kandy / Muruthalawa	Female	46
EPI_ISL_18472774	Asia / Sri Lanka / Kandy / Peradeniya / Gampola	Male	52
EPI_ISL_18472775	Asia / Sri Lanka / Kandy / Peradeniya	Male	38
EPI_ISL_18472776	Asia / Sri Lanka / Kandy	Female	35
EPI_ISL_18472777	Asia / Sri Lanka / Kandy / Yatihalagala	Female	24
EPI_ISL_1847277	Asia / Sri Lanka / Kandy / Peradeniya / Doluwa	Male	27

8			
EPI_ISL_18472779	Asia / Sri Lanka / Kandy	Female	41
EPI_ISL_18472780	Asia / Sri Lanka / Kandy / Handessa	Male	87
EPI_ISL_18472781	Asia / Sri Lanka / Kandy / Peradeniya	Male	37
EPI_ISL_18472806	Asia / Sri Lanka / Kandy	Male	25
EPI_ISL_18472845	Asia / Sri Lanka / Kandy / Peradeniya	Female	25
EPI_ISL_18472846	Asia / Sri Lanka / Kandy / Peradeniya / Gelioya	Male	55
EPI_ISL_18472847	Asia / Sri Lanka / Kandy / Peradeniya / Dangolla	Female	19
EPI_ISL_18472848	Asia / Sri Lanka / Kandy	Male	26
EPI_ISL_18472849	Asia / Sri Lanka / Kandy / Peradeniya	Male	32
EPI_ISL_18472860	Asia / Sri Lanka / Kandy / Kadugannawa	Male	Not known
EPI_ISL_18472862	Asia / Sri Lanka / Kandy	Male	49
EPI_ISL_18472864	Asia / Sri Lanka / Kandy / Peradeniya / Weligalla	Male	60
EPI_ISL_18472866	Asia / Sri Lanka / Kandy / Muruthalawa	Female	81
EPI_ISL_18472871	Asia / Sri Lanka / Kandy / Watapuluwa	Male	38
EPI_ISL_18472892	Asia / Sri Lanka / Kandy / Peradeniya / Pililmathalawa	Male	62
EPI_ISL_18472924	Asia / Sri Lanka / Kandy / Werellagama	Male	29
EPI_ISL_18472925	Asia / Sri Lanka / Kandy / Peradeniya	Female	61
EPI_ISL_18472946	Asia / Sri Lanka / Kandy / Peradeniya / Doluwa	Male	39
EPI_ISL_18472947	Asia / Sri Lanka / Kandy / Handessa	Male	34
EPI_ISL_18472948	Asia / Sri Lanka / Kandy / Uduwela	Female	18
